# Acidic growth conditions stabilize the ribosomal RNA gene cluster and extend lifespan through noncoding transcription repression

**DOI:** 10.1111/gtc.13089

**Published:** 2023-12-08

**Authors:** Yo Hasegawa, Hiroyuki Ooka, Tsuyoshi Wakatsuki, Mariko Sasaki, Ayumi Yamamoto, Takehiko Kobayashi

**Affiliations:** ^1^ Laboratory of Genome Regeneration Institute for Quantitative Biosciences (IQB) The University of Tokyo Bunkyo‐ku Japan; ^2^ Department of Biological Sciences, Graduate School of Science The University of Tokyo Bunkyo‐ku Japan; ^3^ Department of Life Science and Technology Tokyo Institute of Technology Midori‐ku Japan; ^4^ Department of Industrial System Engineering Hachinohe College Hachinohe Japan; ^5^ Collaborative Research Institute for Innovative Microbiology The University of Tokyo Bunkyo‐ku Japan; ^6^ Present address: Laboratory of Gene Quantity Biology National Institute of Genetics Mishima Japan

**Keywords:** acidity, blackcurrant, genome stability, histone deacetylase, lifespan, noncoding transcription, ribosomal RNA gene (rDNA), senescence

## Abstract

Blackcurrant (*Ribes nigrum* L.) is a classical fruit that has long been used to make juice, jam, and liqueur. Blackcurrant extract is known to relieve cells from DNA damage caused by hydrogen peroxide (H_2_O_2_), methyl methane sulfonate (MMS), and ultraviolet (UV) radiation. We found that blackcurrant extract (BCE) stabilizes the ribosomal RNA gene cluster (rDNA), one of the most unstable regions in the genome, through repression of noncoding transcription in the intergenic spacer (IGS) which extended the lifespan in budding yeast. Reduced formation of extrachromosomal circles (ERCs) after exposure to fractionated BCE suggested that acidity of the growth medium impacted rDNA stability. Indeed, alteration of the acidity of the growth medium to pH ~4.5 by adding HCl increased rDNA stability and extended the lifespan. We identified *RPD3* as the gene responsible for this change, which was mediated by the RPD3L histone deacetylase complex. In mammals, as inflammation sites in a tissue are acidic, DNA maintenance may be similarly regulated to prevent genome instability from causing cancer.

## Graphical abstract

We show that blackcurrant extract (BCE) stabilizes the ribosomal RNA gene cluster (rDNA) through repression of noncoding transcription within rDNA intergenic spacers. We found that BCE alters the acidity of the growth medium to pH ~4.5. This growth condition could be linked to an increase in both rDNA stability and yeast replicative lifespan, which was also extended in cells exposed to BCE. We identified RPD3 as the gene responsible for these changes.

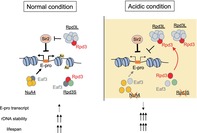

## 1 INTRODUCTION

Blackcurrant (*Ribes nigrum* L.) is a classical fruit that has long been used to make juice, jam, and liqueur in European countries. Blackcurrant, a deciduous shrub, produces a dark purple fruit containing high levels of polyphenols including anthocyanins (Gopalan et al., 2012). Recent studies have confirmed several health benefits conferred by the berries including their antioxidant, anticarcinogenic, anti‐inflammatory, and antimetabolic syndrome properties (Cortez & Gonzalez de Mejia, 2019). Blackcurrant extract (BCE) is known to increase the cells' resistance to DNA damage by oxidant genotoxic reagents, such as hydrogen peroxide (H_2_O_2_), methyl methane sulfonate (MMS), and ultraviolet (UV) radiation (Kato et al., 2012). In budding yeast, genome instability manifested as loss of heterozygosity, was reduced by addition of BCE to the medium (Kato et al., 2012). In mammalian cells, the in vitro Comet assay to assess DNA fragmentation and a micronucleus test, used to screen for genotoxic compounds, indicate that BCE effectively reduces DNA damage (Yamamoto et al., 2017). The BCE activity that protects the genome is not reduced by heat treatment of the extract (Yamamoto et al., 2014). In spite of these findings, the mechanism by which BCE reduces DNA damage is not well understood.

**FIGURE 1:**
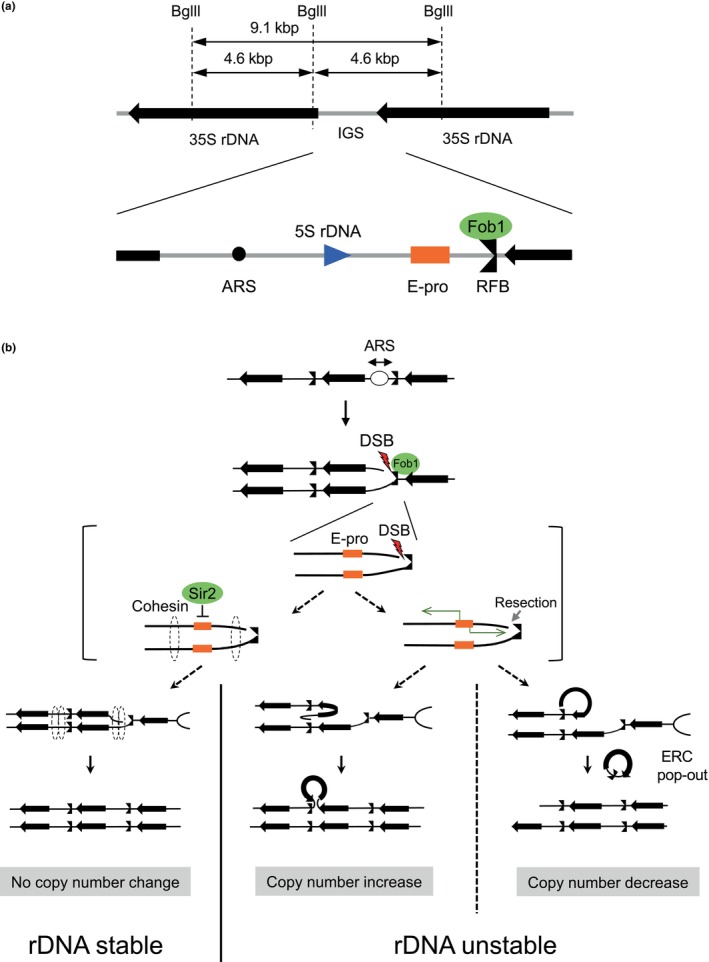
Structure and recombination of rDNA in budding yeast. (a) Schematic diagram of the rDNA locus of budding yeast. Two of the ~150 35S rDNA genes are depicted separated by an intergenic spacer (IGS) containing the 5S rRNA gene, an autonomously replicating sequence (ARS), E‐pro, and RFB site. (b) Schematic of recombination in rDNA. When E‐pro is repressed by Sir2, DSBs caused by Fob1 are repaired in situ due to cohesin association and the rDNA copy number does not change (bottom left). When E‐pro is active, cohesin dissociates and DSB repair can occur by unequal recombination between sister chromatids. Resection facilitates this recombination, which results in copy number variation (bottom middle and right); recombination within the same chromatid produces ERCs (bottom right).

The ribosomal RNA gene (rDNA) is one of the most fragile sites in the genome (Hori et al., 2023; Kobayashi, 2011). The cellular mechanism regulating rDNA instability is well studied, so that the manner how BCE contributes to genome stability can be analyzed by focusing on this region. Eukaryotic rDNA genes are clustered in tandem repeats on the chromosome and recombination between repeats increases genome instability, for example, when this leads to a loss of rDNA copies (Kobayashi, 2008). Cells need many rDNA copies to meet the huge demand for ribosomes and therefore maintain a fixed copy number (Iida & Kobayashi, 2019). A deletion in the rDNA gets restored by a process involving gene amplification (Hawley & Marcus, 1989; Kobayashi et al., 1998). In this mechanism, the unique replication fork barrier (RFB) site, which can be specifically bound by protein Fob1, has a critical role in budding yeast. The RFB site is located at the termination region of the 35S rRNA gene (35S rDNA) to avoid collision between replication and rDNA transcription (Figure 1a). In the S phase of the cell cycle, bidirectional replication starts from a replication origin (ARS, autonomously replicating sequence) in the intergenic spacer (IGS) and the fork propagating against the direction of 35S rDNA transcription is arrested at the RFB site when this is occupied by Fob1 (Figure 1b). Breakage of single‐stranded regions of an arrested fork can lead to a double strand break (DSB) in need of repair (Burkhalter & Sogo, 2004; Kobayashi et al., 2004; Weitao et al., 2003) (Figure 1b). When the copy number is normal (Figure 1b, left panel), Sir2 represses the noncoding promoter E‐pro and a ring‐shaped protein complex, cohesin, holds the sister chromatids together, facilitating DNA repair in situ (Kobayashi & Ganley, 2005). In cells with a reduced rDNA copy number, however, *SIR2* expression is also reduced, and E‐pro is activated. This activation disrupts the cohesin complex which promotes unequal sister‐chromatid recombination that can lead to an increase in copy number (Figure 1b, center) or a decrease (Figure 1b, right). Resection of the DSB end also facilitates recombinational repair (Sasaki & Kobayashi, 2017). Therefore, the rDNA goes through cycles of contraction (due to loss of copies) and amplification (recovery of copies) and becomes an unstable, fragile site. This instability can be monitored by determining the ERC level, that is, the amount of rDNA molecules excised by recombination (Figure 1b, right).

The stability of rDNA has an impact on the lifespan in budding yeast (Ganley et al., 2009; Ganley & Kobayashi, 2014; Hattori et al., 2022; Sinclair & Guarente, 1997). The lifespan of the *fob1* mutant, in which the rDNA is stable by lack of RFB activity and concomitant DSB formation, is extended by ~60% (Defossez et al., 1999 ； Takeuchi et al., 2003). In contrast, the lifespan of the *sir2* mutant, with unstable rDNA due to enhanced unequal sister‐chromatid recombination, is shortened by ~50% compared with wild type (Kaeberlein et al., 1999; Saka et al., 2013).

As mentioned above, the rDNA is sensitive to DNA damage, and its fragility affects lifespan. Moreover, rDNA instability is linked to the formation of ERCs, which can be quantified. Therefore, we decided to analyze the effect of BCE on cell growth and genome stability by focusing, for the latter, on the rDNA. We found that BCE stabilized the rDNA and extended yeast replicative lifespan. Analysis of various BCE fractions showed that acidity could be a factor causing these effects. We found that mildly acidic growth conditions (pH ~4.5) were sufficient to stabilize rDNA by repression of noncoding transcription in the IGS and to extend the lifespan. We identified Rpd3 and other proteins of the RPD3L histone deacetylase complex to be responsible for acid‐dependent rDNA stabilization. Rpd3 was found to associate with the IGS and involved in reducing the synthesis of noncoding transcripts at pH 4.5. These findings suggest that the acidity of the environment is an important factor in maintaining genome stability and thereby in controlling yeast lifespan.

## 2 RESULTS

### 2.1 Exposure to blackcurrant extract increases rDNA stability

**FIGURE 2:**
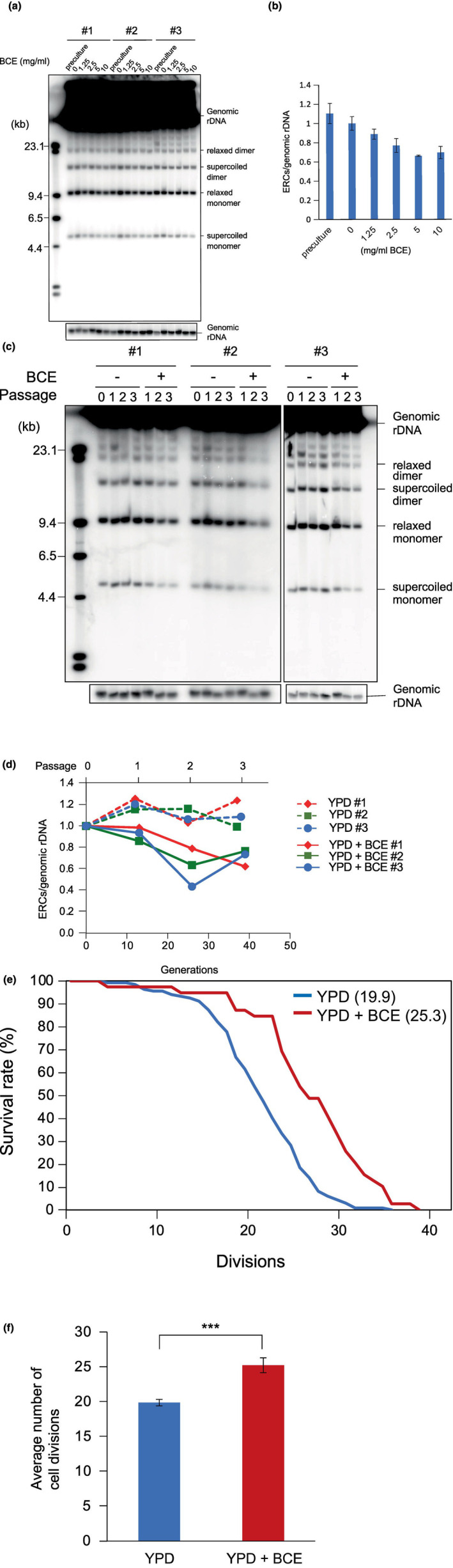
Exposure to blackcurrant extract stabilizes rDNA and extends lifespan. (a,b) ERC levels in cells exposed to blackcurrant extract (BCE). ERCs in cells grown on media with indicated concentrations of BCE were detected by Southern blotting (a). During gel electrophoresis, supercoiled ERC exhibits a more compact structure and migrates at a higher speed compared with the relaxed form, which contains nicks in the DNA. A monomer consists of one unit of rDNA, while a dimer consists of two. Three independent experiments were performed. The signal intensities were normalized to those of genomic rDNA (short exposure, bottom panel) (b). Pearson's product rate correlation coefficient from 0 to 5 mg/mL BCE was calculated to be *ρ* = −.74, a negative correlation. (c,d) BCE in the medium reduces ERC formation in long‐term cultures. Cells taken from the pre‐culture (Passage 0) were propagated on medium with and without BCE, and their ERC levels were detected by Southern blotting (c). ERC levels were quantified relative to those in the pre‐culture and the short exposure of genomic rDNA (bottom panel) (d). Three independent experiments were performed; the number of passaging steps and generations are indicated. (e,f) Presence of BCE in the growth medium extended lifespan. Survival curves (e) and average number of cell divisions (f) were determined by counting the number of daughter cells that budded from a mother cell on YPD (*n* = 135) and in the presence of BCE (*n* = 39). In (e), average number of cell divisions from f is shown within parentheses. Statistical significance of observed differences was assessed by Student's *t*‐test; ***means *p* < .001.

Blackcurrant extract (BCE) makes cells resistant to DNA damage (Yamamoto et al., 2014). We analyzed whether BCE had an effect on the stability of the rDNA, which is a fragile site in the genome (Kobayashi, 2011). As a marker for rDNA stability, we monitored the level of ERCs, which are circular rDNA molecules that pop out from the cluster by recombination (Figure 1b). In cells cultured in the presence of various concentrations of BCE, the number of ERCs were detected by gel electrophoresis followed by Southern blotting (Figure 2a). ERC signals were quantitated relative to the genomic rDNA (Figure 2b) which showed that ERC levels were decreasing as the concentration of BCE increased to 5 mg/mL. Comparable ERC levels at the higher BCE concentration of 10 mg/mL indicated that 5 mg/mL BCE in the medium suffices to stabilize rDNA. This concentration was used in subsequent experiments.

To determine whether exposure to BCE has a long‐term effect on rDNA stability, we continued the liquid culture of yeast by repeated dilution. As shown in Figure 2c, in all three independent experiments, ERC levels were reduced in cells cultured in BCE‐containing medium for ~40 generations. Exposure to BCE for only 10 generations was sufficient to reduce ERC amounts by ~30% in comparison to cultures not growing in the presence of BCE (Figure 2d). At 25 and 40 generations the negative effect of BCE on ERC formation became more obvious. These results indicate that 5 mg/mL BCE in the medium is sufficient to permanently reduce cellular ERC levels, thereby indicating that BCE exposure increases rDNA stability.

### 2.2 Exposure to blackcurrant extract extends yeast lifespan

Addition of BCE to the growth medium increases the resistance of cells to DNA damage (Yamamoto et al., 2014). DNA damage is known to promote senescence and to reduce lifespan in many organisms (Schumacher et al., 2021). In budding yeast, ERC accumulation in the mother cell restricts the replicative lifespan, that is, the number of cell divisions of the cell (Sinclair & Guarente, 1997). Therefore, to test the effect of BCE on yeast replicative lifespan, we determined the number of cell divisions on agar plates with and without BCE. Due to the size‐difference between parental cells and their offspring, the replicative lifespan could be measured by counting the number of buds that detached from a cell under a microscope (Kennedy et al., 1994). The results are shown as survival curves (Figure 2e) and the average number of cell divisions (Figure 2f). Consistent with the observation that the presence of BCE in the medium has a DNA‐stabilizing effect, the yeast replicative lifespan on a BCE‐containing plate was extended by ~27%.

### 2.3 Acidic growth conditions enhance rDNA stability and lifespan

**FIGURE 3:**
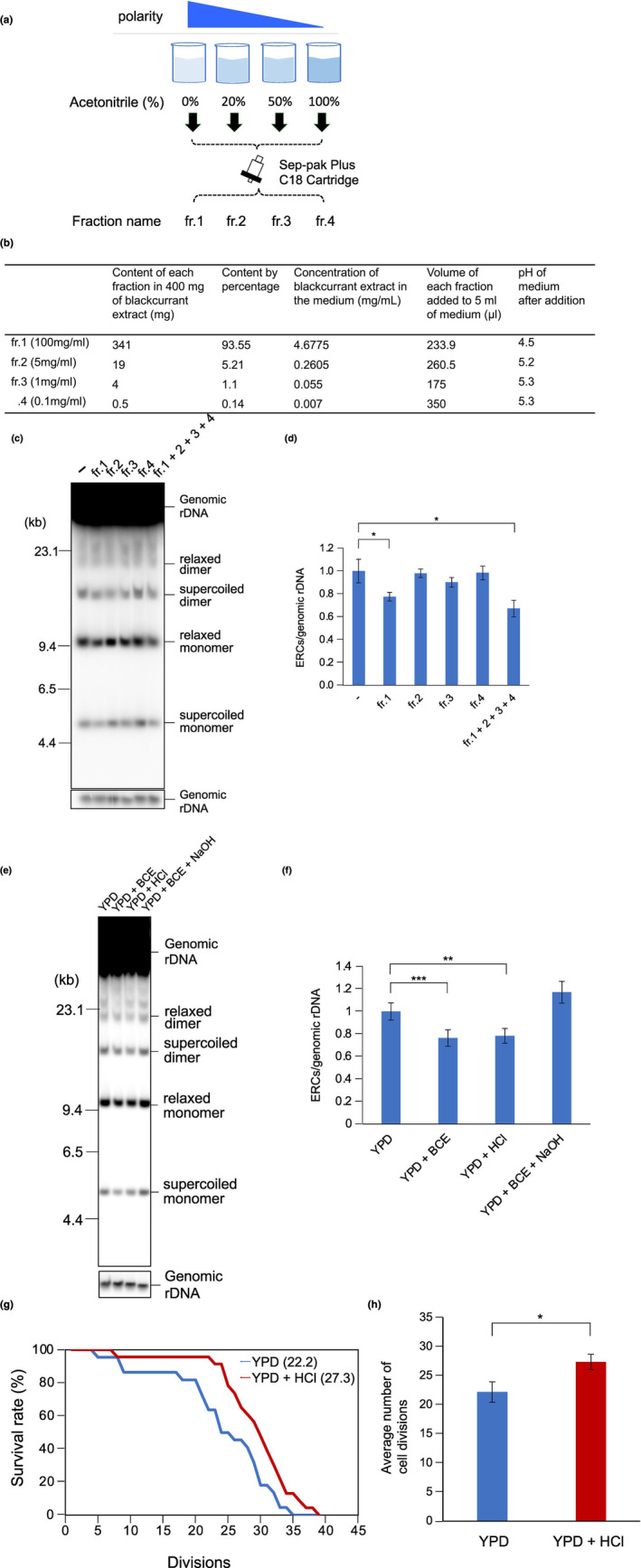
Analysis of blackcurrant extract after fractionation. (a) Schematic diagram of BCE fractionation by reversed‐phase chromatography. BCE was divided into four fractions according to the polarity of the mixture of water and acetonitrile (fr.1 to fr.4). (b) The mass of the components in each fraction was determined after lyophilization. From these results, the percentage and mass of each fraction in the blackcurrant extract were calculated. (c,d) ERC formation in cells cultured on medium supplemented with indicated fractions was detected by Southern blotting (c) and quantitated as in Figure 2d. Between (−) and fr.1, *p* value is .018. Between (−) and fr.1 + 2 + 3 + 4, *p* value is .017. (e) Growth under acidic conditions lead to a reduction in ERC formation. ERCs in cells grown in YPD, YPD + BCE, YPD + HCl (pH ~4.5) and YPD + BCE + NaOH (pH ~6.1) were detected by Southern analysis. (f) Quantification of Southern analysis in e. The error bars show mean ± s.e.m (*n* = 3). Between YPD and YPD + BCE, *p* « .001. Between YPD and YPD + HCl, *p* = .0047. (g,h) Lifespan increase after adjustment of the media to pH 4.5. Survival curves (g) and average number of cell divisions (h) were determined for cells cultured on YPD (pH 6.1, *n* = 22, blue line) and with added HCl (pH 4.5, *n* = 23, red line) plates. In G, average number of cell divisions from h is shown within parentheses. *p* = .023. Statistical significance of observed differences was checked with Student's *t*‐test.

To determine the factor that affects rDNA stability and lifespan, we analyzed the effect of BCE on ERC formation after fractionation. In short, the extract was bound to a hydrophobic column (Sep‐pak Plus C18) and eluted with 0%, 20%, 50%, and 100% acetonitrile yielding four fractions (Figure 3a). The four fractions (Figure 3b) were tested for their effect on rDNA stability by assaying the ERC levels in cells growing on media supplemented with material derived from each fraction (Figure 3c). As shown in Figure 3d, the main activity to reduce ERC formation resided in the hydrophilic flow‐through (fr.1). An outstanding feature of this fraction was its acidity (pH ~4.5) (Figure 3b). Therefore, we tested whether acidity by itself could have an effect on rDNA stability by adding HCl instead of BCE to the medium so that the pH was reduced to ~4.5. Quantitation of ERC signals in the Southern analysis of Figure 3e showed a comparable reduction of ERC levels in cells growing in media supplemented with HCl or BCE (Figure 3f). In contrast, after raising the pH of BCE‐containing media to 6.1 by addition of NaOH, no stabilization of rDNA was detected in cells cultured in this medium (YPD + BCE + NaOH). Moreover, there was correlation between acidity and the amount of ERC (Figure S1). Therefore, we concluded that a pH of 4.5 is a growth condition that leads to reduced ERC formation and contributes to maintain rDNA stability with normal growth rate.

Interestingly, the amount of ERC was less when the medium pH was adjusted with acetic acid than when with HCl (Figure S2). Acetic acid is known to have some other cellular functions that change pH of vacuole and activate mitochondrial function (Carmelo et al., 1997; Hughes & Gottschling, 2012). Therefore, we used HCl‐adjusted medium to analyze the effect of medium acidity for further study.

In view of the effect of pH on DNA damage (Figure 3f) and that of BCE on lifespan (Figure 2e,f), we expected that the replicative lifespan of cells would depend on the pH of their growth conditions. To test this, we measured the lifespan of cells incubated on plates adjusted with HCl to pH ~4.5 and compared this with the lifespan of cells growing on normal plates (pH ~6.1). The survival curves (Figure 3g) and the average number of cell divisions (Figure 3h) showed an extension of the lifespan by 23% when the environmental pH is ~4.5. Taken together, our results indicate that growth conditions of low acidity (pH 4.5) increase rDNA stability and extends lifespan.

We have to note that the previous study showed medium acidity does not affect replicative lifespan. The reason could be the use of different yeast strain (BY4742) and acidity (pH 5.0 by HCl) (Wasko et al., 2013). Especially, we think acidity (pH. ~ 4.5) is important to reduce ERC and extend lifespan (Figure S2).

### 2.4 Noncoding IGS transcription is repressed in cells cultured at pH 4.5

To obtain insights into the mechanism how environmental acidity contributes to maintaining rDNA stability, we examined factors known to induce rDNA damage such as DSBs. This kind of rDNA damage occurs when replication forks are blocked by Fob1 bound to the RFB site. First, we compared the RFB activity between cells grown at pH 4.5 and at normal pH by two‐dimensional gel electrophoresis. This technique can visualize replication intermediates (Figure S3a–c). DNA was isolated from cells in the early logarithmic growth phase and digested with restriction enzyme BglII. RFB activity was estimated from the intensity of the spot (Arrested forks) that corresponds to the accumulation of replication intermediates arrested at the RFB (Brewer et al., 1992; Kobayashi et al., 1992). As shown in Figure S3BC, the intensities of replication forks arrested at the RFB (arrows) were similar for cells cultured at pH ~6.1 (YPD) and those grown at pH ~4.5 (YPD + HCl), thereby indicating that the RFB activity is not affected by acidity of the growth medium. Next, we compared the occurrence of DSBs that trigger recombination and are indicative for rDNA instability. For this purpose, we separated the same DNA fragments as used in Figure S3B by normal gel electrophoresis. By Southern analysis, we could detect DNA fragments (~2.2 kb) that correspond to molecules broken at the RFB (Burkhalter & Sogo, 2004; Kobayashi et al., 2004; Weitao et al., 2003). The signal intensities of these bands were normalized to that of the band representing the replication forks arrested at the RFB, which showed that DSB formation was comparable between cells grown under normal (YPD) or acidic (YPD + HCl) conditions (Figure S3d,e). As a control, rDNA was analyzed that had been isolated from *fob1*mutant cells. These cells lack RFB activity, and the associated DSBs would not be generated (lower panel, extended exposure). Taken together, these results showed that growth under acidic conditions has no effect on RFB activity and the formation of DSBs (Figures S3c,e).

**FIGURE 4:**
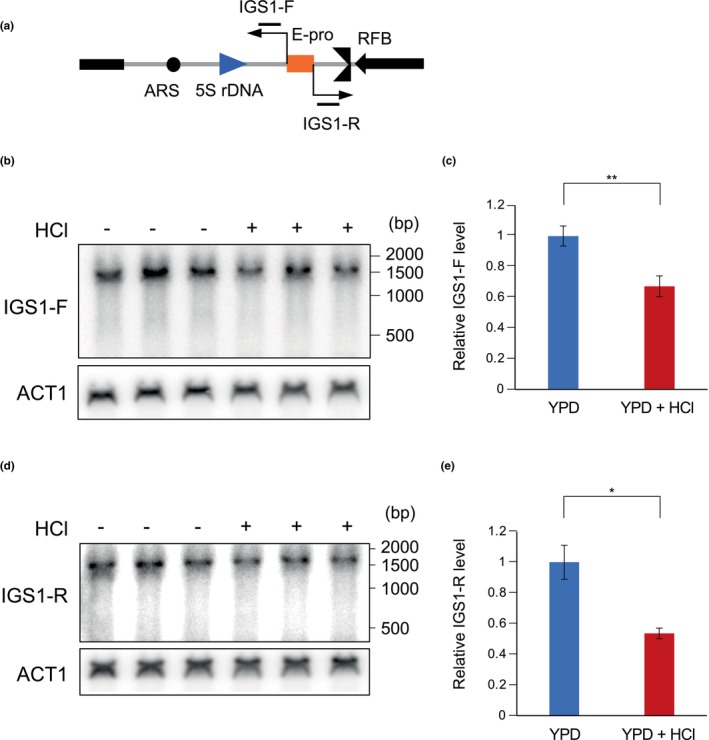
Acid‐dependent repression of E‐pro. (a) Positions of probes to detect transcripts from E‐pro are indicated by black lines. (b–e) Transcripts of E‐pro from the wild type grown in YPD and YPD + HCl (pH ~4.5) medium were detected by Northern blotting. Blots were hybridized with IGS1‐F (b) and IGS1‐R probes (d). Membranes were stripped and rehybridized to a ACT1 probe as a control for loading. The signal intensities normalized to those of *ACT1* are shown (c,d). The error bars show mean ± s.e.m (*n* = 3). In IGS1‐F, *p* = .025. In IGS1‐R, *p* = .036. Statistical significance of differences was assessed by Student's *t*‐test.

Noncoding transcription from E‐pro in the IGS (Figures 1b and 4a) enhances unequal sister‐chromatid recombination and increases rDNA instability (Kobayashi & Ganley, 2005). To assess whether this process could be affected by acidity of the environment, RNA was isolated from cultures incubated under normal (YPD) or acidic conditions with the pH adjusted to ~4.5 (YPD + HCl) and analyzed by Northern blotting (Figure 4b,d). To detect noncoding RNA transcribed in either direction, we used two single‐stranded probes, IGS1‐F and IGS1‐R (Figure 4a). The transcript signals detected with these probes were normalized to that of ACT1 (Figure 4c,e), which showed that addition of HCl to the growth medium caused reduced transcription in either direction. These results suggested that noncoding transcription is affected by growth under acidic conditions which leads to an increase of the rDNA stability.

### 2.5 Histone (de)acetylases involved in repression of IGS transcription at pH 4.5

**FIGURE 5:**
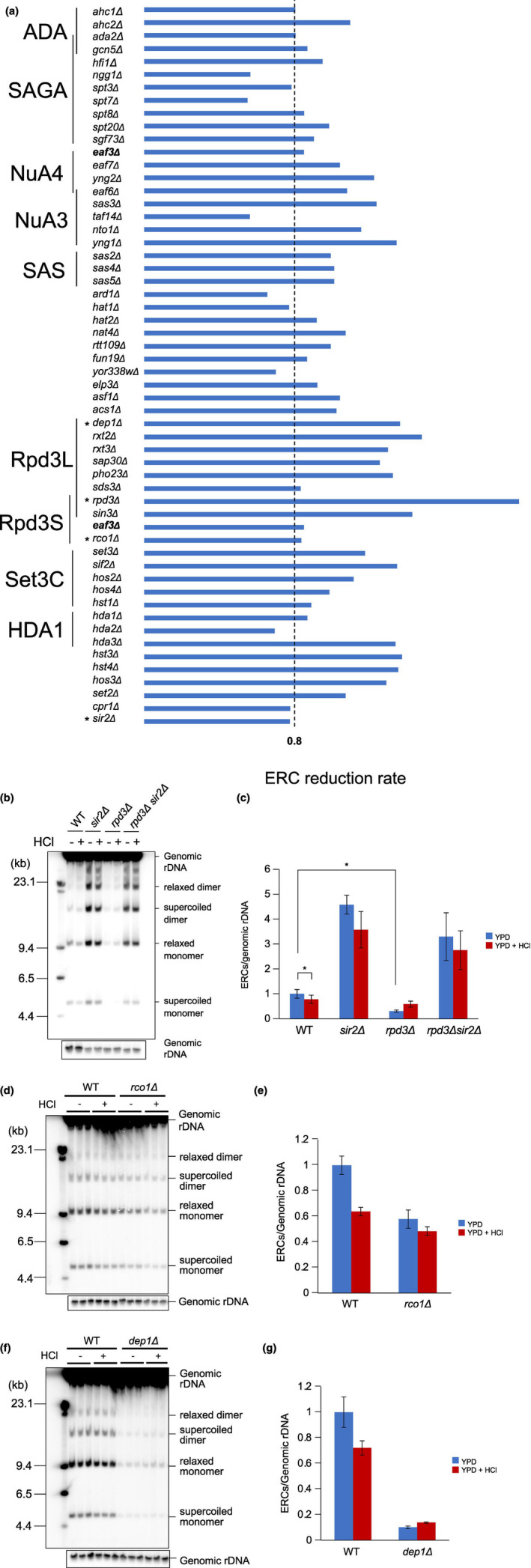
Acid‐dependent ERC reduction in histone modification mutants. (a) ERC reduction rates for mutants carrying a deletion of a histone (de)acetylation related gene. The dashed line (0.8) indicates the rate of the wild‐type strain (See text for details). Screening was performed on deletion strains in a BY4741 genetic background, except for *rpd3Δ*, *dep1Δ*, *rco1Δ*, and *sir2Δ* mutants that were made in W303 (*); *eaf3Δ* (bold) is involved in both NuA4 and Rpd3S complexes. (b,c) Acid‐dependent ERC reduction depends on Rpd3. ERCs were detected in the *rpd3Δ*, *sir2Δ*, and the double mutants by Southern analysis (b) and quantified (c) as described for Figure 2. The error bars shown mean ± s.e.m (*n* = 3); *indicates *p* < .05. (d–g) Acid‐dependent ERC reduction in *rco1Δ* and *dep1Δ* mutants compared with wild type. ERC levels detected by Southern analysis (d,f) under normal (YPD, −) and acidic growth conditions (YPD + HCl, +) were quantitated (e,g) as described for Figure 2. The error bars show mean ± s.e.m (*n* = 3).

Gene(s) responsible for increased rDNA stability in cells cultured at pH 4.5 can be expected to have an impact on noncoding transcription from the E‐pro, which was the sole factor to be altered under acidic conditions (Figure 4). Therefore, we turned our attention to (de)acetylase genes involved in histone modification which are important for the regulation of transcription in general. From a yeast deletion library, 55 mutants were picked and tested for loss of the ability to stabilize rDNA in an acidic environment by comparing ERC levels in cells cultured at pH 6.1 (control) with those grown on acidic medium at pH 4.5. For this, each mutant was characterized by an ERC reduction rate calculated as the ratio between ERC levels under acidic versus control conditions. This rate was ~0.8 for the wild type, meaning that the amount of ERCs is reduced by ~20% when cells are exposed to acidic growth conditions. Mutants whose reduction rate exceeds 0.8 are candidates that have lost the ability to stabilize rDNA under acidic conditions. As shown in Figure 5a, for over 40 mutants, the rate was beyond 0.8, indicating reduced repression of ERC production under acidic growth conditions. In other words, many histone modification enzymes are active during growth in an acidic environment in ERC production. The rate for the *rpd3Δ* mutant was increased the most, to ~2. Therefore, we further analyzed the role of the Rpd3 histone deacetylase.

The ERC data for the *rpd3Δ* mutant are shown in Figure 5b and quantified in Figure 5c. As reported previously (Yoshida et al., 2014), ERC levels are significantly reduced in the absence of Rpd3 in general. This might be related to reduced replication initiation, a process Rpd3 is known to regulate (Yoshida et al., 2014). In addition, we also confirmed the rDNA stability in the chromosome increases by pulsed‐field gel electrophoresis in which the rDNA stability is detected by the shape of chromosome XII that has rDNA (Figure S4) (Kobayashi et al., 1998). As shown in the figure, the bands in the *rpd3Δ* mutant were sharper than those in the wild type. Therefore, recombination frequency itself is also reduced in the *rpd3Δ* mutant.

Under acidic conditions, the amount of ERCs relatively increased in *rpd3Δ* cells, while in wild‐type cells, these amounts were reduced. Sir2 affects rDNA stability, and in its absence, ERC levels raise significantly under normal growth conditions but decrease at a rate comparable to wild type (Figures 5a) when cells are exposed to pH 4.5 (Figure 5b,c). Compared with *sir2Δ*, in the *rpd3Δ sir2Δ* double mutant, ERC levels are slightly reduced under normal and acidic conditions while the ERC reduction rate did not appear to change, thereby suggesting that Rpd3 and Sir2 functions in the same pathway in ERC formation.

Rpd3 is known to be part of two complexes, Rpd3S and Rpd3L (Rundlett et al., 1996), which have distinct roles in the regulation of gene expression (Biswas et al., 2008). Rco1 is an essential component of Rpd3S and Dep1 is specific to Rpd3L (Johnson et al., 2013). We assessed ERC levels in deletion mutants for these genes (Figure 5d–g) and calculated their ERC reduction rate (Figure 5a). Compared to wild type, ERC levels were generally reduced in the *rco1Δ* mutant, while under acidic conditions, these levels decreased but to a lesser extent (Figure 5d,e). In contrast, the *dep1Δ* mutant showed different phenotypes from the *rco1Δ* mutant. As shown in the Figure 5f,g, the amount of ERCs was reduced, and in the acidic condition, it was increased as observed for the *rpd3Δ* mutant (Figure 5b,c). This suggests that it is the Rpd3L complex that negatively contributes to rDNA stability in general (see discussion). Moreover, in the *dep1Δ* mutant, there was no reduction of ERC levels under acidic growth conditions, which suggests that Rpd3L activity contributes to maintaining rDNA stability at pH 4.5.

### 2.6 Rpd3 regulates rDNA stability and lifespan through noncoding transcription

**FIGURE 6:**
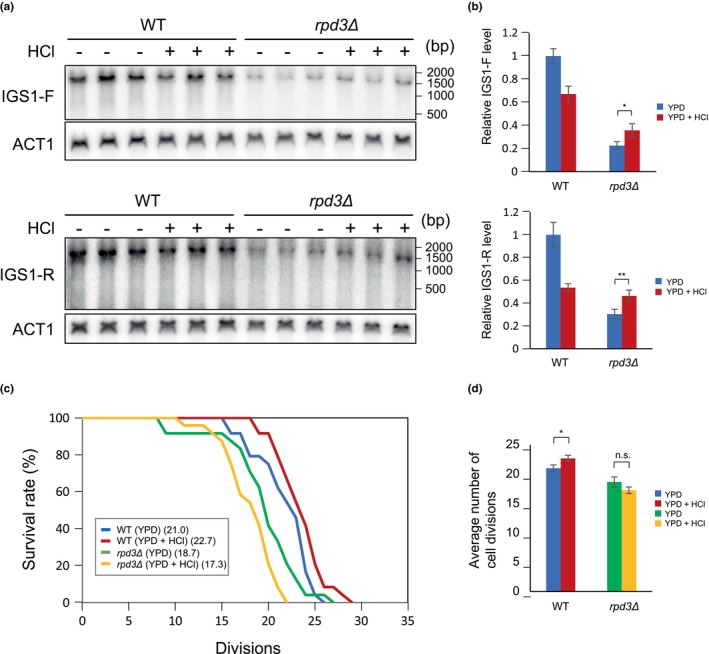
Acid‐dependent E‐pro repression and lifespan extension is lost in *rpd3Δ* cells. (a,b) E‐pro repression in the *rpd3Δ* mutant and wild type was assessed by Northern blot analysis of RNA isolated from cells grown under acidic (YPD + HCl, +) and normal (YPD, −) conditions (a). IGS‐F and IGS‐R signals were quantified (b) as described for Figure 4, with *p* = .038 (*) and *p* = .006 (**) for the difference between transcript levels in *rpd3Δ* cells. (c,d) Lifespan analysis of wild‐type and *rpd3Δ* cells grown under acidic and normal conditions. Survival curves (c) and the average number of cell divisions (d) were determined. The difference between the average number of wild‐type cell divisions (WT) at pH ~6.1 versus pH ~4.5 is significant (*p* = .028).

Finding Rpd3 to be required for reducing ERC levels in cells grown under acidic conditions, we tested whether this histone deacetylase regulated transcription from the E‐pro in the IGS, which is reduced under such conditions (Figure 4). In contrast to wild‐type cells, in the *rpd3Δ* mutant, IGS transcription in either direction was slightly elevated at pH 4.5 (YPD + HCl, +) compared with normal conditions (YPD, −) (Figure 6a,b). Therefore, Rpd3 appears to be responsible for acid‐dependent reduction of E‐pro activity. As observed for ERC formation (Figure 5b,c), total transcription from E‐pro was much lower in the absence of Rpd3 than in wild‐type cells.

In line with the observations that both suppression of ERC formation and E‐pro transcription under acidic conditions depended on the presence of Rpd3, we expected that lifespan extension at pH 4.5 would be affected in the *rpd3Δ*. Indeed, as shown in Figure 6c,d, *rpd3Δ* cells had a shorter lifespan than wild‐type cells, even more so at pH 4.5, mirroring the reversal in ERC formation (Figure 5b,c) as expressed by the ERC reduction rate (Figure 5a). These results suggest that Rpd3 is a factor that senses or initiates a response to acidity in the medium and thereby regulates replicative lifespan.

## 3 DISCUSSION

Blackcurrant is a fruit that has long been used to make juice, jam, and liqueur. Though blackcurrant looks like a blueberry, it is too sour (acid) to eat without cooking and not so popular as blueberries for our daily diet. Blackcurrant is known as a healthy food with high antioxidants, such as anthocyanin and vitamin C (Gopalan et al., 2012; Oczkowski, 2021; Zafra‐Stone et al., 2007) and the extract relieves DNA damage and stabilizes the genome in vivo (Yamamoto et al., 2014). How the extract contributes to genome stability is poorly understood. Here we report our finding that the acidity of the extract is one of the factors that help to maintain the genome, especially the rDNA that is one of the largest fragile sites. By genetic analysis, we identified many genes that are involved in acid‐dependent stabilization of the rDNA. One of them was *RPD3* that codes for a histone deacetylase (Rundlett et al., 1996). In the *rpd3Δ* mutant, the acid‐dependent rDNA stabilization disappeared. We found that the noncoding transcription in the IGS that increases rDNA instability is repressed under acidic conditions and the repression disappears in the *rpd3Δ* mutant. As a result, the rDNA is more stabilized which extends lifespan under acidic conditions in the wild type strain, but not in the *rpd3Δ* mutant. Our findings provide more evidence that acidity contributes to our health through maintenance of genome stability.

We tested 55 histone modification enzymes as candidates for acid‐dependent rDNA stabilizing factors because these could be expected to be involved in the control of noncoding transcription in the IGS, which was repressed in cells cultured under acidic conditions. We think that most of the 40 genes that were found to be required for increased ERC‐suppression under acidic growth conditions could be involved in reducing transcription from the E‐pro as observed in the case of *RPD3* when cells were exposed to pH 4.5.

**FIGURE 7:**
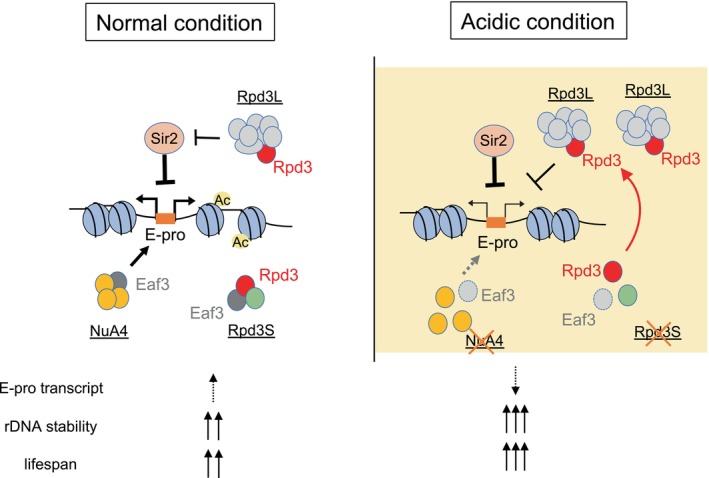
Model for E‐pro repression under acidic growth conditions. Under normal growth conditions, Sir2, a histone deacetylase, represses E‐pro and thereby maintains rDNA stability, which is counteracted by the NuA4 histone acetylase complex. Under acidic growth conditions, Eaf3 loses the association with the NuA4 and Rpd3S complexes, which dissociate. The released Rpd3 moves to the Rpd3L complex so that more Rpd3L can deacetylate histones around E‐pro. This leads to a tighter repression of noncoding transcription. Moreover, dissociation of NuA4 also reduces E‐pro expression. As a result, rDNA is more stabilized and the lifespan is extended under acidic growth conditions.

The phenotype of the *rpd3Δ* mutant is puzzling. As reported previously, the rDNA is silenced in this mutant (Smith et al., 1999), which reflects the general reduction in ERC formation and E‐pro transcription we observed (Figures 5b,c, 6a,b). These results, which may relate to a reduction of replication initiation activity, could mean that Rpd3 is a positive transcription activator in spite of its histone deacetylase activity. On the other hand, Rpd3 acts as a negative regulator inducing repression of transcription (from the E‐pro) when cells are exposed to acidic growth conditions, which would fit its enzymatic activity. Furthermore, Figure 5b,c demonstrates that Sir2 and Rpd3 functioned in the same pathway in ERC formation. One plausible explanation is that Rpd3 serves as a negative regulator of Sir2 under the normal condition (as illustrated in Figure 7, ‘Normal condition’), possibly by inhibiting Sir2's association with the E‐pro. Consequently, in the *rpd3Δ* mutant, Sir2 functions with greater efficiency, leading to reduced ERC formation and decreased E‐pro transcription (Figures 5b,c, 6a,b).

While, in the acidic condition, the reduction in ERCs can be attributed to the activation of Rpd3 as a histone deacetylase that represses E‐pro transcription, as shown in Figure 6a,b. Moreover, it is known that Rpd3 is a subunit of different complexes (Rpd3S and Rpd3L) and the distribution of Rpd3 over these complexes changes in response to exposure to an acidic environment. The Rpd3L complex represses the E‐pro in an acid‐dependent manner (Figure 5a–c,f,g). Rpd3 is associated with Eaf3 in the Rpd3S complex, while Eaf3 is also a component of the NuA4 histone acetylase complex (Figure 5a) (Wakatsuki et al., 2019). Complex formation of Eaf3 is known to be reduced in cells with low intracellular pH (Okuda & Nishimura, 2020), so that Eaf3 can be expected to dissociate from the Rpd3S complex under the acidic condition and the member Rpd3 is released to form the Rpd3L that silences the E‐pro (Figure 7, right).

As for the reason for increase of E‐pro transcription in the *rpd3Δ* mutant (Figure 6a,b), we simply speculate that these is no reduction of E‐pro repression by the active Rpd3. Further study is required to test these models and reveal the mechanism at play.

In this research, we used the budding yeast as a model. Blackcurrant extract (BCE) also makes mammalian cells resistant to DNA damage (Yamamoto et al., 2014), so that a similar genome stabilization may be expected due to acidity of the cellular surroundings. What is the biological meaning of enhanced genome stability in eukaryotic cells exposed to an acidic environment? In the case of budding yeast, cells live on the surface of fruits, such as grapes and strawberries. Therefore, their living place is somehow acidic and the yeast has adapted to it.

In the case of mammals, the pH of body fluid is ~7.4, whereas a low pH is observed around inflammation sites in mammalian tissue (Riemann et al., 2015). In the inflammation sites, DNA damage may occur more frequently because of wounded cells, active cell divisions to heal, and the production of ROS (Canli et al., 2017; Grivennikov et al., 2010). An elevated tolerance to DNA damage will be beneficial to cells in inflamed tissue to reduce cancer risk, pointing to the possibility that an acid‐driven genome maintenance system could be conserved between yeast and mammalian cells.

## 4 EXPERIMENTAL PROCEDURES

### 4.1 Budding yeast strains

Strains derived from the W303 laboratory strain of the budding yeast *Saccharomyces cerevisiae* were used in this study, as listed in Table S1.

Most strains used in the screening of Figure 5a came from the Yeast Knock Out Strain Collection (Funakoshi).

### 4.2 Preparation of BCE (blackcurrant extract)

To 80 g of blackcurrant fruit (Aomori blackcurrant association), 400 mL of RO water was added and set in a fiber mixer (Panasonic MX‐X300‐K) and crushed with high‐speed rotation for 10 s at room temperature. After crushing, the sample was aliquoted into centrifuge tubes and centrifuged at 10,000 × g for 10 min using a Beckman Coulter JA‐14 rotor. The supernatant was filtered through a coffee filter, centrifuged for another 10 min as above, and filtered through a coffee filter again after which the BCE was sterilized through a 0.2 μm filter and lyophilized. For lyophilization, 15 mL of the BCE was dispensed into 50 mL Falcon tubes, which were placed on Styrofoam covered with ice and left overnight at −80°C. The next day, the lid of the Falcon tube was removed, covered with aluminum foil aseptically, and five to six holes were punched through the aluminum foil with a toothpick before placing the tubes into the lyophilizer. After lyophilization, the extracts were weighed and dissolved in sterile water to 100 mg/mL and stored at −20°C.

### 4.3 Fractionation of BCE by reversed‐phase chromatography

Reversed‐phase chromatography with a mixture of water and acetonitrile was performed on the BCE. A C18 Plus column (Sep‐Pak) was used as stationary phase and as mobile phases mixtures of sterile water and acetonitrile: 100%: 0% (sterile water), 80%: 20% (mixed solvent 1), 50%: 50% (mixed solvent 2), and 0%: 100% (acetonitrile).

The C18 column was prepared as follows: using a 10 mL syringe (Terumo), the column was washed twice with 5 mL of sterile water, then with 2 mL of solvent mixture 1, 5 mL of solvent mixture 2, two washes with 5 mL of acetonitrile, and finally with 5 mL of sterile water twice.

Using a 10 mL syringe, 1 mL of BCE diluted 10‐fold with sterile water was passed through a prepared C18 column, and 1 mL of the eluate was collected as fraction 1 (designated as fr.1). Next, two washes with 5 mL of sterile water were passed through the C18 column, and the eluates were combined with fraction 1. Then, 2 mL of solvent mixture 1 was passed through the C18 column and the eluate was collected as fraction 2 (fr.2). The eluate from the next wash with 5 mL of solvent mixture 2 yielded fraction 3 (fr.3). Then two washes with 5 mL of acetonitrile were eluted and combined to fraction 4 (fr.4). Finally, 5 mL of sterile water was passed through the C18 column twice and discarded as waste.

The above series of operations were repeated to fractionate in total 4 mL of 100 mg/mL BCE. An evaporator was used to reduce the solvent in the four fractions which were then lyophilized, weighed, and dissolved in sterile water to 100 mg/mL (fr. 1), 5 mg/mL (fr.2), 1 mg/mL (fr.3), and 0.1 mg/mL (fr.4), and stored at −20°C.

### 4.4 Yeast cultures

Yeast cultures were grown in YPD liquid medium (yeast extract 1% [wt/vol], Bacto peptone 2% [wt/vol], glucose 2% [wt/vol]), in YPDA (YPD containing a final concentration of 40 μg/mL adenine) and on YPD agar medium (YPD with Bacto agar 2% [wt/vol]) at 30°C. BCE was added to YPD medium to a final concentration of 5 mg/mL. For analysis of fractionated BCE, the medium was prepared as shown in Figure 3b. Routinely, yeast cultures were inoculated from pre‐cultures that had been growing for ~18 h at 30°C and prepared by dispersing single colonies, scraped from a YPD plate that had been incubated for two days after streaking cells from a frozen stock, into 5 mL of medium. For high‐acidity YPD liquid medium, the pH was adjusted using hydrochloric acid (HCl) or acetic acid (CH_3_COOH) (min. 99.0% [wt/wt] (Wako)). After pH adjustment, the medium was sterilized by autoclaving, and the pH at room temperature was measured with a pH meter.

YPD agar was found not to coagulate under acidic conditions (Kanazawa & Kunito, 2012). Therefore, in an experiment to measure yeast longevity using acidic medium (Figures 3g and 6c), 2x YPD medium which was adjusted to pH 4.5 using hydrochloric acid and 2x agar water (Bacto Agar 4% [wt/vol]) were prepared separately and autoclaved and equal amounts were mixed just prior to pouring the plates. The pH of the agar medium was checked by placing a portion on pH test paper (Advantec universal).

For the detection of ERCs, cell counts of the pre‐culture were determined using a microscope and flasks with pH‐adjusted and pH‐unadjusted medium were inoculated at a cell concentration of 2 × 10^4^ cells/ml and incubated until cell counts reached 1.0 to 1.25 × 10^8^ cells/ml. Then, 1 × 10^8^ cells were collected and 5 × 10^7^ cells were used for the preparation of each of two agarose plugs with genomic DNA.

For assessing the effective BCE concentration of Figure 2a, cell counts of pre‐cultures in YPD were determined and 5 × 10^7^ cells were collected for electrophoresis, while the remainder was used to inoculate YPD medium without (0 mg/mL) or with BCE (at concentrations of 1.25, 2.5, 5, and 10 mg/mL) to a cell density of 2 × 10^4^ cells/ml and cultured for 18 h. After incubation, 5 × 10^7^ cells were collected from each culture for ERC analysis. A similar approach was used to determine the effect of long‐term exposure to BCE on rDNA stability (Figure 2c,d). Cells from a pre‐culture were harvested (Passage 0) as well as diluted and further incubated with or without 5 mg/mL BCE for 18 h (Passage 1). These steps were repeated for each culture for two subsequent growth cycles (Passage 2 and Passage 3).

RNA for the analysis of E‐pro transcription was isolated from cells grown in 20 mL of YPDA on a shaking incubator shaker (Innova) at 210 rpm after the OD600 (measured with an Ultrospec 2100 pro; GE Healthcare) had increased ~4‐fold from a starting density of 1.3 × 10^6^ cells/mL.

For experiments measuring RFB activity and DSB frequency, cell counts were measured in YPD pre‐cultures and pH‐adjusted and pH‐unadjusted medium was inoculated to cell concentrations of 2 × 10^4^ cells/mL and 1 × 10^8^ to 1.25 × 10^8^ cells/mL, respectively, and incubated for 2–3 h. Cells were diluted into pH‐adjusted and pH‐unadjusted medium to a density of 1 × 10^6^ cells/ml and further incubated until the OD600 had doubled. The cells were collected in 50 mL tubes and sodium azide was added to a final concentration of 0.1% to kill the cells.

### 4.5 Electrophoresis of genomic DNA

DNA plugs for ERC analysis, pulsed‐field gel electrophoresis, 2D gel electrophoresis, and DSB analysis were prepared as previously described (Kobayashi et al., 1998; Sasaki & Kobayashi, 2017; Yanagi et al., 2022). As for pulsed‐field gel electrophoresis, one‐third of a plug was loaded on 1.0% agarose gel (pulsed‐field Certified Agarose, Bio‐Rad) in 0.5× TBE buffer (44.5 mM Tris base, 44.5 mM boric acid and 1 mM EDTA, pH 8.0). The electrophoresis conditions were: 68 h at 3.0 V/cm at 14°C, 120° included angle, initial switch time of 300 s, and final switch time of 900 s by Bio‐Rad CHEF Mapper. Gel electrophoresis for the analysis of ERCs (Yanagi et al., 2022) and the detection of DSB activity (Yanagi et al., 2022) were performed as described previously, while 2D gel electrophoresis to assess RFB activity was as described (Yanagi et al., 2022) with the following modifications. Plugs, cut to about 3 mm, were incubated in 300 μL FastDigest buffer (Thermo Fisher Scientific) for 20 min at room temperature. The FastDigest buffer was replaced and the plugs were incubated for another 20 min, after which 250 μL of FastDigest buffer and 5 μL of FastDigest BglII (Thermo Fisher Scientific) were added and the incubation was continued overnight at 37°C. After the reaction, the plugs were washed with 50 mM EDTA (pH 7.5), placed on a comb, and solidified with 0.4% agarose, 1× TBE (89 mM Tris base, 89 mM boric acid, 2 mM EDTA pH 8.0) in a 15 × 20 cm tray. Then, 1.5 L of 1× TBE was poured into the electrophoresis tank, the gel was placed, and 175 ng of Hind III digested λ DNA was applied as a size marker. DNAs were separated in the first dimension by electrophoresis for 14 h, at 1.0 V/cm, at room temperature. After electrophoresis was completed, the gel was stained with 0.5 μg/mL ethidium bromide (EtBr) and photographed under UV light. A region ranging from 2.1 to 9.4 kbp was cut out, rotated 90°, and solidified with 1.2% agarose, 1× TBE in a 15 × 20 cm tray. After pouring 1.5 L of 1× TBE into the electrophoresis tank and placing the gel, DNAs were separated in the second dimension by electrophoresis for 6 h, 5.0 V/cm at 4°C.

### 4.6 Southern blot analysis to assay ERC levels, RFB activity, and DSB frequency

After electrophoresis, gels were treated with 0.5 M hydrochloric acid and denatured in 1.5 M sodium chloride, 0.5 M sodium hydroxide. DNA was transferred to Hybond‐XL (GE healthcare) by capillary action using 1.5 M sodium chloride, 0.25 M sodium hydroxide. After transfer, the membrane was treated with 0.4 M sodium hydroxide for 10 min and air‐dried. The probe used for the detection of genomic rDNA and ERCs was prepared as previously described (Hosoyamada et al., 2019).

For ERC detection, membranes were exposed to a phosphor screen, and the scanned images were used to quantify various ERC forms (relaxed dimer, supercoiled dimer, relaxed monomer, and supercoiled monomer), following the previously established method (Hosoyamada et al., 2019). The frequency of ERCs was determined by calculating the ratio of the ERC signal to the genomic rDNA signal in each lane.

### 4.7 Northern blot analysis to detect the E‐pro transcript

Preparation of probes and Northern analysis was done as previously described (Yanagi et al., 2022). In ultrasensitive hybridization buffer (Thermo Fisher Scientific), probes IGS1‐F and IGS1‐R were incubated with the membrane to detect transcripts derived from E‐pro after which the membrane was stripped in boiled 0.1% SDS for 30 min and washed with 2× SSC, 0.1% SDS before the membrane was re‐hybridized to the ACT1 probe.

### 4.8 Measurement of replicative life span

This analysis was performed as previously described (Yanagi et al., 2022).

## Supporting information


**Figure S1.** Acidity affects the amount of ERC. (A,B) ERC was detected in different acidic medium. Southern blotting (A) and the bands were quantitated as in Figure 2. Lane 1: pH 6.1, lane 2: pH 5.7, lane 3: pH 5.5, lane 4: pH 5.2, lane 5: pH 4.8, lane 6: pH 4.4, lane 7: ph 3.8, lane 8: pH 3.4, lane 9: pH 2.9. The acidity of medium was adjusted with hydrochloric acid. The error bars show mean ± s.e.m (*n* = 3). Statistical significance of observed differences was assessed by Student's *t*‐test; *means *p* < .05.
**Figure S2.** Acetic acid reduces ERC production more than HCl. (a) ERC was detected in YPD, YPD + BCE, YPD + HCl (pH ~4.5) and YPD + CH_3_COOH (pH ~4.5) by Southern analysis. (b) Quantification of Southern analysis as in Figure 2. The error bars show mean ± s.e.m (*n* = 3). Statistical significance of observed differences was assessed by Student's *t*‐test; *means *p* < .05.
**Figure S3.** RFB and DSB activities were not affected by acidic condition. (a) Schematic of the signal pattern of two‐dimensional gel electrophoresis. (b) Replication intermediates of rDNA in acid (YPD + HCl) and nonacidic (YPD) conditions. The signal intensities of arrested replication forks (Arrested forks) that correspond with RFB activity are similar in both acid and nonacidic conditions. (c) Quantitation of replication forks arrested at the RFB (arrows). The signal intensities were normalized to those of replication intermediates. The error bars show mean ± s.e.m (*n* = 3). (d) DSB activity at the RFB in acid and nonacidic conditions. The signal intensities of DSB bands (DSB) were detected in acid (HCl +) and nonacidic (HCl −) conditions. The *fob1* mutant is a negative control that does not have RFB and DSB activities. The bottom panel is a long exposure of DSB signals. (E) Quantification of DSB activity. The signal intensities of DSB bands were normalized to those of the replication forks arrested at the RFB. The error bars show mean ± s.e.m (*n* = 3); n.s. means the difference was not statistically significant by *t*‐test. The lane with the size marker (M) is shown.
**Figure S4.** rDNA stability in *sir2Δ*, *rpd3Δ*, and the double mutant rDNA stabilities in the *rpd3Δ, sir2Δ*, and the double mutants grown in nonacidic condition were detected by pulsed‐field gel electrophoresis. The mutants were prepared by tetrad analysis. The chromosomal size marker is *Hansenula wingei* (Bio‐Rad).
**Table S1.** Strain list used in this study.
