# Supplementary material for: Acidic growth conditions stabilize the ribosomal RNA gene cluster and extend lifespan through noncoding transcription repression
Source: Genes Cells. 2023 Dec 8;29(2):111–30. doi: 10.1111/gtc.13089 (PMC11447830; doi:10.1111/gtc.13089)
Supplement: Supplementary file 1 — Figure S1. Acidity affects the amount of ERC. (A,B) ERC was detected in different acidic medium. Southern blotting (A) and the bands were quantitated as in Figure 2. Lane 1: pH 6.1, lane 2: pH 5.7, lane 3: pH 5.5, lane 4: pH 5.2, lane 5: pH 4.8, lane 6: pH 4.4, lane 7: ph 3.8, lane 8: pH 3.4, lane 9: pH 2.9. The acidity of medium was adjusted with hydrochloric acid. The error bars show mean ± s.e.m (n = 3). Statistical significance of observed differences was assessed by Student's t‐test; *means p < .05. Figure S2. Acetic acid reduces ERC production more than HCl. (a) ERC was detected in YPD, YPD + BCE, YPD + HCl (pH ~4.5) and YPD + CH3COOH (pH ~4.5) by Southern analysis. (b) Quantification of Southern analysis as in Figure 2. The error bars show mean ± s.e.m (n = 3). Statistical significance of observed differences was assessed by Student's t‐test; *means p < .05. Figure S3. RFB and DSB activities were not affected by acidic condition. (a) Schematic of the signal pattern of two‐dimensional gel electrophoresis. (b) Replication intermediates of rDNA in acid (YPD + HCl) and nonacidic (YPD) conditions. The signal intensities of arrested replication forks (Arrested forks) that correspond with RFB activity are similar in both acid and nonacidic conditions. (c) Quantitation of replication forks arrested at the RFB (arrows). The signal intensities were normalized to those of replication intermediates. The error bars show mean ± s.e.m (n = 3). (d) DSB activity at the RFB in acid and nonacidic conditions. The signal intensities of DSB bands (DSB) were detected in acid (HCl +) and nonacidic (HCl −) conditions. The fob1 mutant is a negative control that does not have RFB and DSB activities. The bottom panel is a long exposure of DSB signals. (E) Quantification of DSB activity. The signal intensities of DSB bands were normalized to those of the replication forks arrested at the RFB. The error bars show mean ± s.e.m (n = 3); n.s. means the difference was not statistically signif [file GTC-29-111-s001.pdf]

Supplemental Fig.S1

A

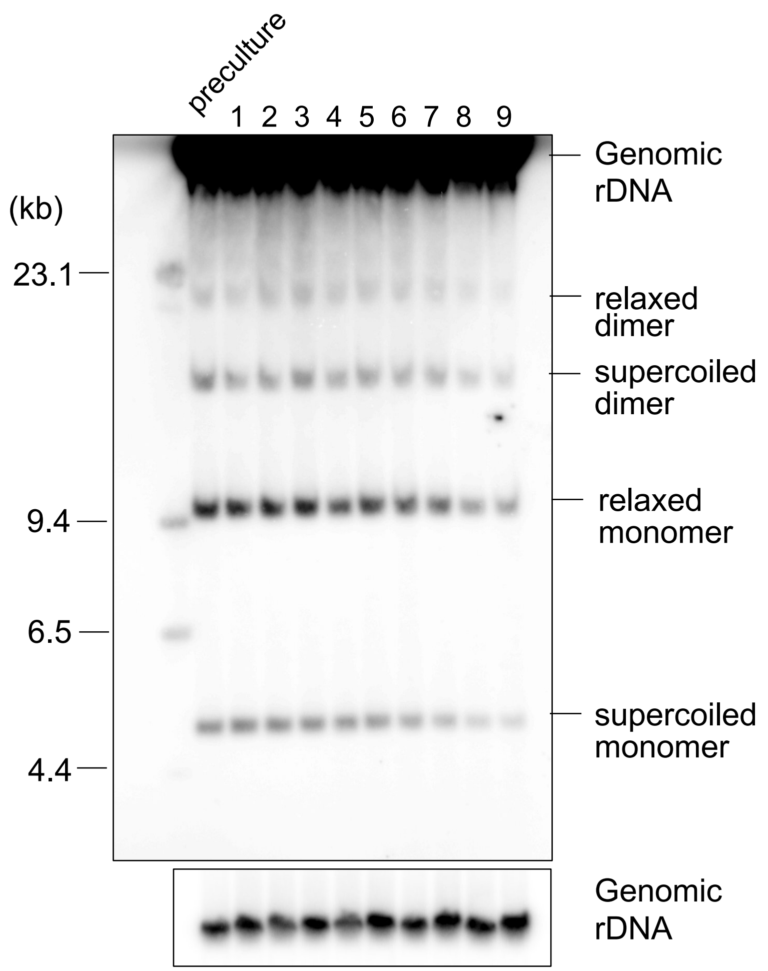

B

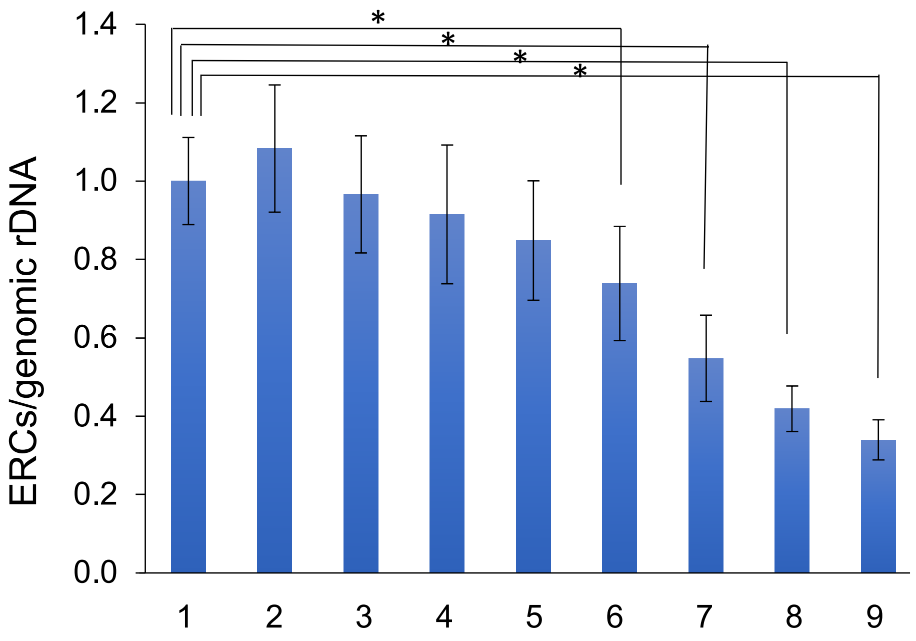

Supplemental Fig.S2

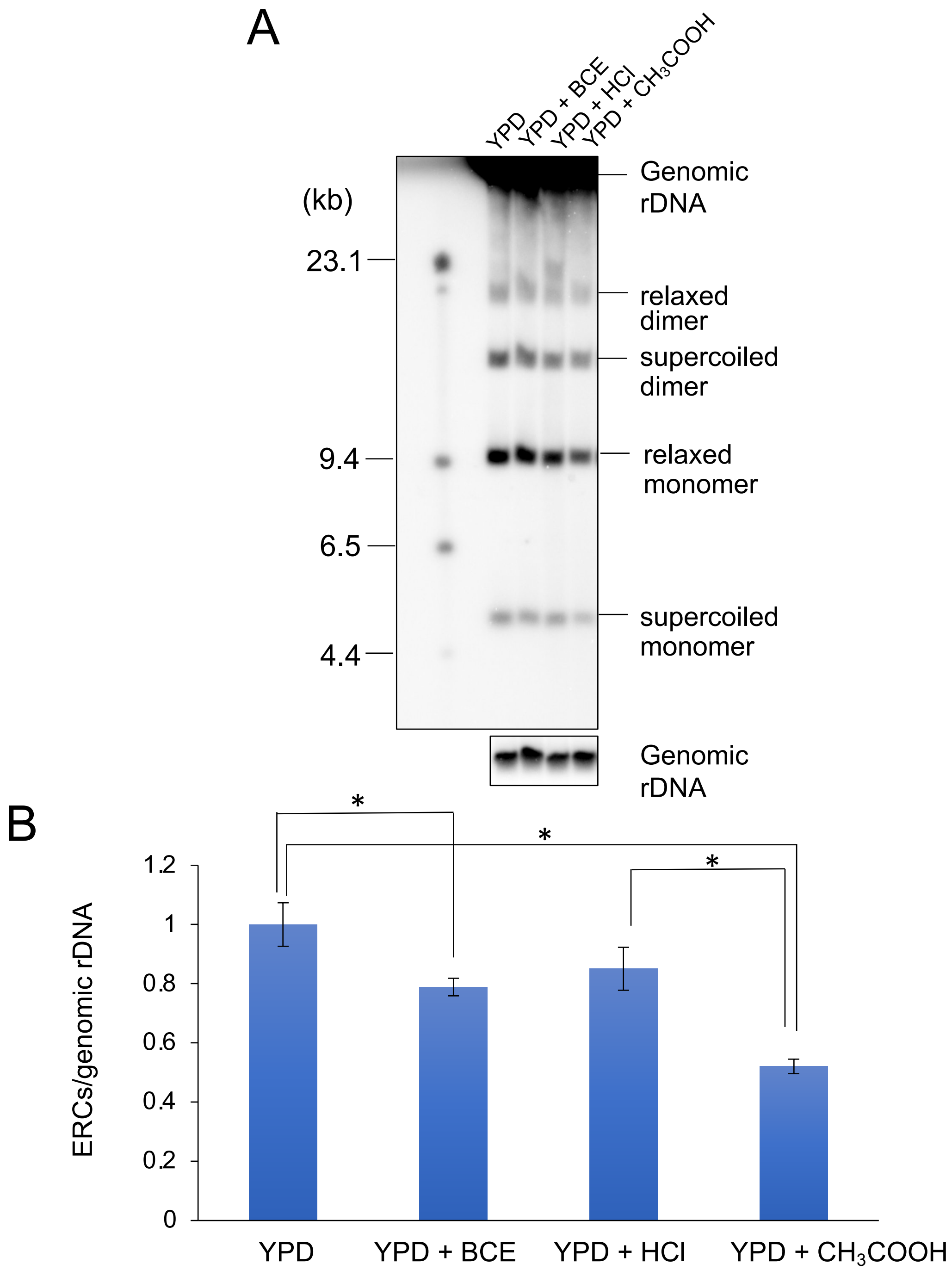

Supplemental Fig.S3

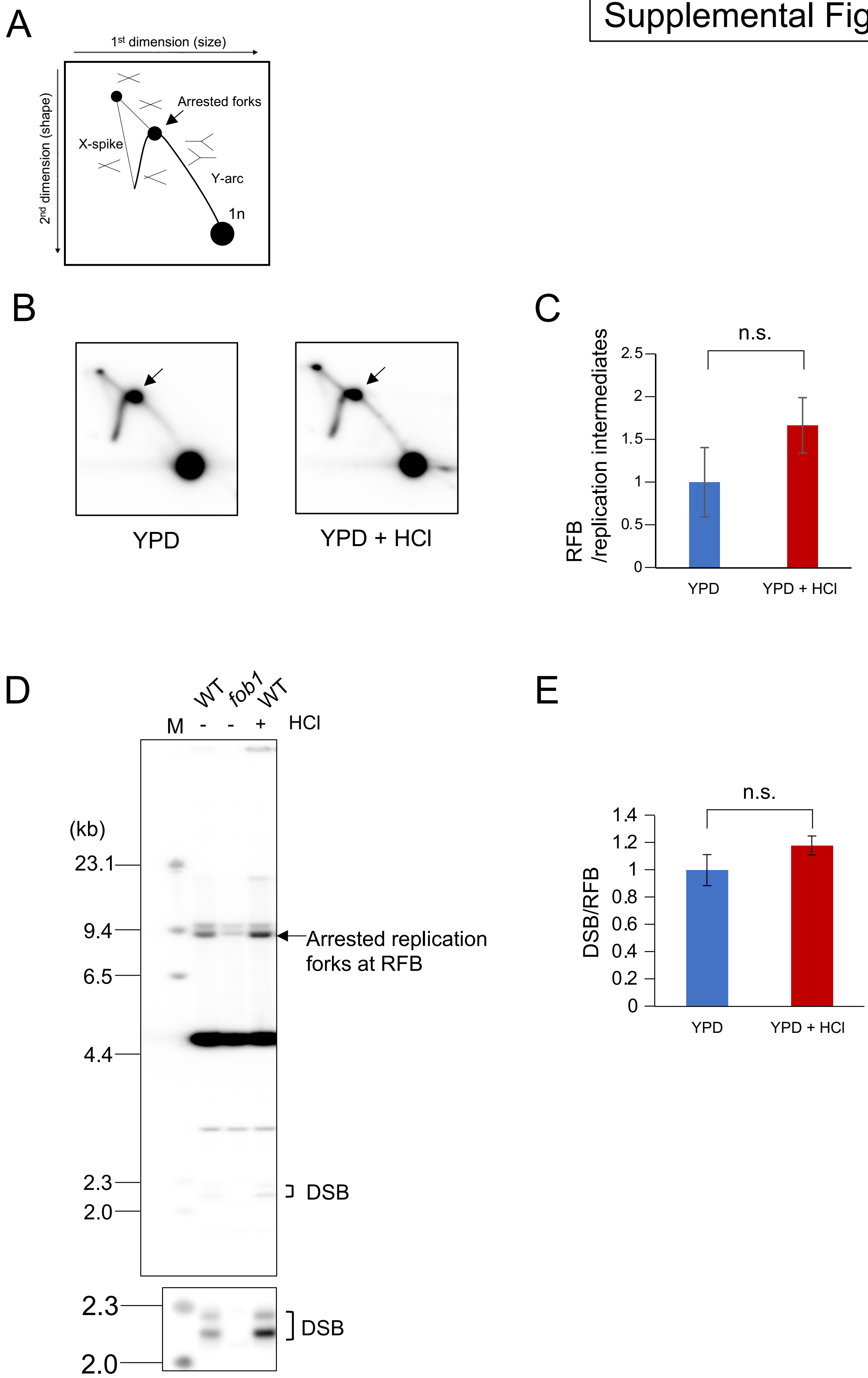

Supplemental Fig.S4

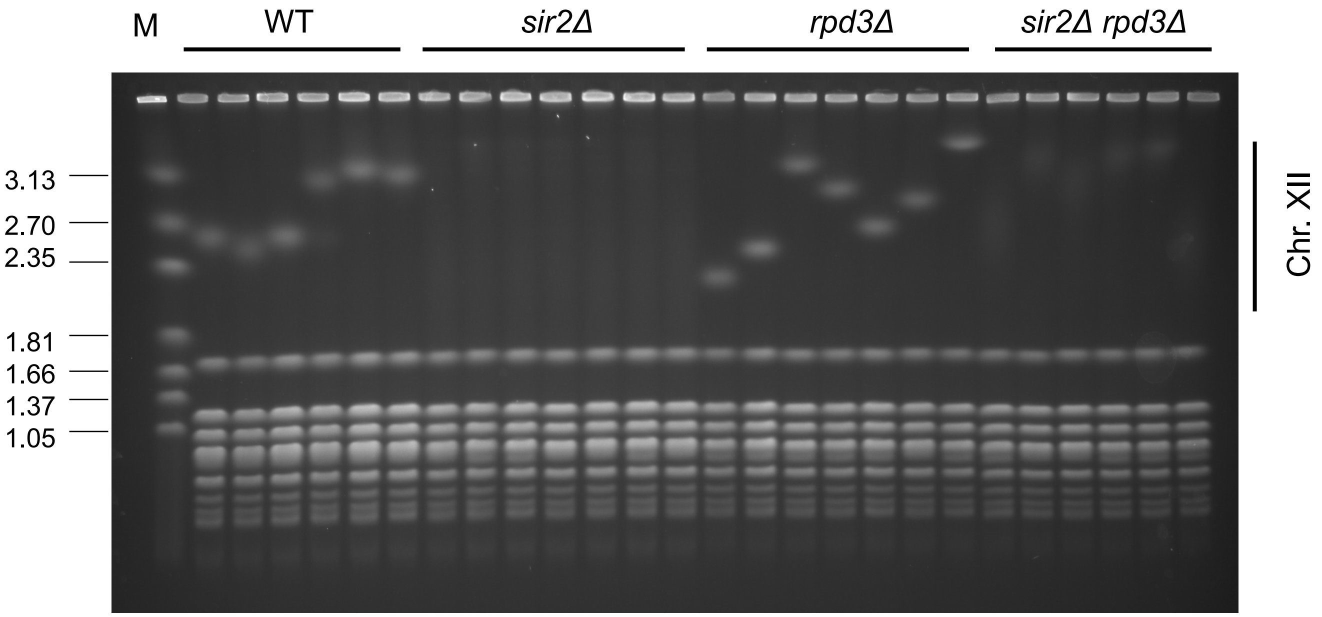

Supplemental Table S1

Table S1 Strain list used in this study.

Strain	Genotype	Background
**Wild-type**	*MATa, ade2-1, ura3-1, his3-11,15, trp1-1, leu2-3,112, can1-100*	W303
fob1	*MATa, ade2-1, ura3-1, his3-11,15, trp1-1, leu2-3,112, can1-100, fob1::LEU2*	W303
sir2Δ	*MATa, ade2-1, ura3-1, his3-11,15, trp1-1, leu2-3,112, can1-100, sir2Δ::kanMX*	W303
rpd3Δ	*MATa, ade2-1, ura3-1, his3-11,15, trp1-1, leu2-3,112, can1-100, rpd3Δ::hphMX*	W303
dep1Δ	*MATa, ade2-1, ura3-1, his3-11,15, trp1-1, leu2-3,112, can1-100, dep1Δ::kanMX*	W303
rco1Δ	*MATa, ade2-1, ura3-1, his3-11,15, trp1-1, leu2-3,112, can1-100, rco1Δ::kanMX*	W303

Strain used in Figure 5A came from the Yeast Knock Out Strain Collection (Funakoshi).
